# Echocardiographic patterns in treatment-naïve HIV-positive patients in Lagos, south-west Nigeria

**DOI:** 10.5830/CVJA-2012-048

**Published:** 2012-09

**Authors:** DA Olusegun-Joseph, JNA Ajuluchukwu, AC Mbakwem, DA Oke, NU Okubadejo, CC Okany

**Affiliations:** Department of Cardiology, Lagos University Teaching Hospital, Lagos, Nigeria; Department of Cardiology, Lagos University Teaching Hospital, Lagos; Department of Medicine, College of Medicine, University of Lagos, Lagos, Nigeria; Department of Cardiology, Lagos University Teaching Hospital, Lagos; Department of Medicine, College of Medicine, University of Lagos, Lagos, Nigeria; Department of Cardiology, Lagos University Teaching Hospital, Lagos; Department of Medicine, College of Medicine, University of Lagos, Lagos, Nigeria; Department of Cardiology, Lagos University Teaching Hospital, Lagos; Department of Medicine, College of Medicine, University of Lagos, Lagos, Nigeria; Department of Cardiology, Lagos University Teaching Hospital, Lagos; Department of Haematology, College of Medicine, University of Lagos, Lagos, Nigeria

**Keywords:** HIV, cardiac abnormalities, echocardiography

## Abstract

**Introduction:**

Cardiovascular abnormalities are common in HIV-infected patients, although often clinically quiescent. This study sought to identify by echocardiography early abnormalities in treatment-naïve patients.

**Methods:**

One hundred patients and 50 controls with no known traditional risk factors for cardiovascular disease were recruited for the study. The cases and controls were matched for age, gender and body mass index. Both groups had clinical and echocardiographic evaluation for cardiac abnormalities, and CD_4_ count was measured in all patients.

**Results:**

The cases comprised 57 females (57.0%) and 43 males (43.0%), while the controls were 28 females (56.0%) and 22 males (44.0%) (χ^2^ = 0.01; p = 0.913). The mean age of the cases was 33.2 ± 7.7, while that of the controls was 31.7 ± 9.7 (*t* = 1.02; *p* = 0.31). Echocardiographic abnormalities were significantly more common in the cases than the controls (78 vs 16%; *p* = 0.000), including systolic dysfunction (30 vs 8%; *p* = 0.024) and diastolic dysfunction (32 vs 8%; *p* = 0.002). Other abnormalities noted in the cases were pericardial effusion in 47% (χ^2^ = 32.10; *p* = 0.000) and dilated cardiomyopathy in 5% (five); none of the controls had either complication. One patient each had aortic root dilatation, mitral valve prolapse and isolated right heart dilatation and dysfunction.

**Conclusion:**

Cardiac abnormalities are more common in HIV-infected people than in normal controls. A careful initial and periodic cardiac evaluation to detect early involvement of the heart in the HIV disease is recommended.

## Abstract

Human immunodeficiency virus (HIV) possesses an intrinsic cardiopathogenic action that may be detected in even the early stages of HIV disease.^1^ The medical literature clearly documents that HIV/AIDS is a multi-systemic disease, affecting virtually every organ and system of the body, and causing progressive dysfunction.^2^-^3^ It is an established fact that the heart is not spared in the exploits of this rampaging entity.^4^-^6^

Cardiovascular abnormalities are common in HIV-infected patients, although they are often clinically quiescent and frequently attributed to dysfunction in other organ systems.^7^-^9^ Of interest is the observation that the incidence of AIDS-related heart disease found in post-mortem studies is significantly higher than the incidence of abnormalities diagnosed clinically ante mortem.^10^ Therefore it is possible that many AIDS patients have cardiac abnormalities that are not recognised during the course of their illness.

In an autopsy study carried out in 1998, cardiac abnormalities were noted in two-thirds of the patients with AIDS.^11^ These abnormalities, which were attributed directly or indirectly to the HI virus and/or treatment side effects, could largely have been detected early ante mortem using echocardiography, a non-invasive, radiation-free investigation.^10^,^12^,^13^

Cardiac involvement impacts on the natural history and prognosis of the HIV disease. This demands an awareness by clinicians of its cardiovascular manifestations for a complete and rational diagnosis and management.^5^ This study sought to identify echocardiographic abnormalities in treatment-naïve patients in order to assess the cardiac effects of HIV infection, while excluding drug effects.

## Methods

This was a descriptive, cross-sectional study of 100 patients with HIV infection recruited via the HIV clinic of the Lagos University Teaching Hospital (LUTH), Lagos, Nigeria. The patients were yet to commence antiretroviral therapy. The cohort was made up of HIV-infected individuals referred to, or identified in the clinic. They had no prior history of cardiac disease, and were not previously diagnosed as hypertensive or diabetic. Those with a history of use of illicit drugs or previous treatment with drugs with cardiotoxic effects were excluded.

Fifty healthy, HIV-negative individuals served as controls. They were recruited after voluntary screening in the HIV clinic side laboratory to confirm their negative status. Other exclusion criteria for the HIV-positive patients were also applicable in the control group. They were recruited to match the age, gender and body mass index (BMI) profile of the HIV cases.

The research was carried out in accordance with the Declaration of Helsinki. The study protocol was explained to all participants and they gave their informed consent. Approval for the study was obtained from the local Ethics Committee in the institution.

Patients and controls underwent thorough clinical evaluation with an emphasis on the cardiovascular system. Venous blood was collected from all HIV-infected patients for lymphocyte typing to obtain the CD_4_ cell count.

Transthoracic echocardiography was performed using a Siemens Sonoline S1-450 in the cardiovascular laboratory with a 3.5-MHZ transducer probe. Each subject was briefed on the non-invasive nature of the procedure to allay fear and anxiety. Two-dimensional (2D), M-mode, pulse-wave, continuous-wave and colour Doppler echocardiography assessment was done with the subject in the left lateral decubitus position.^12^ The two-dimensional images were obtained in the parasternal longand short-axis views, apical and subcostal views.^12^,^13^

Left atrial diameter (LA), aortic size (AO), right ventricular outflow tract (RVOT), left ventricular end-systolic (LVEDs) and end-diastolic (LVEDd) diameters, interventricular septum (IVS), left ventricular posterior wall (LVPW), estimated right ventricle (ERV), and end-point septal separation (EPSS) measurements were obtained from 2D directed, M-mode recordings from the parasternal long axis.^13^ Measurements were taken (in cm) according to the American Society of Echocardiography guidelines (leading-edge methodology).^14^ The mean of three measurements was recorded.

Doppler studies included pulmonary velocity (PV), aortic velocity (AV), transmitral flow, and deceleration time (DT) measurements. Isovolumetric relaxation time (IVRT) was obtained from pulse-wave Doppler studies.^13^ Echocardiographic abnormalities, e.g. pericardial effusion, thickening, separation, valvular lesions such as stenosis, and regurgitations and regional wall-motion abnormalities were also looked for.

The following definitions were used: dilated left ventricle refers to LVEDd > 5.2 cm.^13^ Left ventricular systolic dysfunction was determined by left ventricular fractional shortening (LVFS) < 28%.^9^,^15^,^16^ The fractional shortening was computed from the basic linear measurements using an appropriate formula:^15^

$LVFS\;=\;\frac{(LVEDd-LVEDs)\;}{LVED\times 100\;}$

The severity of LV dysfunction was graded based on the recommendation by the ESC:^15^ mild dysfunction, fractional shortening = 22–27% ; moderate = 17–21%; severe < 16%. The ejection fraction was calculated using the formula:15

$EF=\;\frac{LVEDV-LVESV\;}{\;LVEDV\;\times 100}$

where LVEDV (left ventricular end-diastolic volume) = LVEDd^3^, and LVESV (left ventricular end-systolic volume) = LVEDs^3^. Dilated cardiomyopathy was diagnosed using three criteria: left ventricular end-diastolic diameter (LVEDd) > 5.5 cm,^16^-^18^ global hypokinesia, and fractional shortening (LVFS) < 28%.^16^,^19^

Isolated right heart dilatation: right ventricle and atrium larger than left ventricle and atrium, respectively on standard two-dimensional echocardiography in apical view; right ventricular end-diastolic dimension > 3.0 cm with normal left ventricular size and function.^18^,^20^

Left ventricular diastolic dysfunction was diagnosed in the presence of any of the following criteria:^21^

• impaired relaxation with an E/A ratio < 1, IVRT > 100 ms and DT > 220 ms
• pseudonormalisation resembling the normal trans-mitral configuration with regard to the mitral inflow but with normal or low DT
• restrictive pattern with E/A ratio > 2, IVRT < 70 ms and DT < 160 ms.

Pericardial effusion refers to an echo-free space behind the left ventricle with or without an anterior echo-free space. The size of the pericardial effusion was defined as follows: small when the maximum pericardial space at end-diastole was < 1.0 cm, moderate when the space was ≥ 1.0 cm but < 2.0 cm, and massive/severe when the pericardial space was ≥ 2.0 cm between the pericardial layers.^22^,^23^

## Results

A total of 100 HIV-positive cases and 50 healthy control subjects were recruited for the study. The cases comprised 57 females (57.0%) and 43 males (43.0%), while the controls included 28 females (56.0%) and 22 males (44.0%). The gender distribution was comparable (χ^2^ = 0.01; *p* = 0.913). The mean age and BMI were not statistically different.

The most common symptoms relevant to the heart were cough (23%), palpitations (11%) and shortness of breath (7%). Most were, however, non-specific as many of the patients had associated anaemia, infections and pulmonary disease, which could have accounted for these symptoms. Only two patients had overt symptoms of heart failure (dyspnoea at rest, orthopnoea, paroxysmal nocturnal dyspnoea, leg swelling, tender hepatomegaly), while one had features of massive pericardial effusion. All three had a CD_4_ count less than 100/μl.

The mean pulse rate was significantly higher in the cases than the controls (87.04 ± 13.04 and 78.56 ± 6.22, respectively; *p* = 0.000) (Table 1). There was no significant difference between the systolic blood pressure (SBP) of the cases and controls. The diastolic blood pressure (DBP) of the cases was, however, significantly lower than that of controls (70.59 ± 7.39 and 74.60 ± 7.27, respectively; *p* = 0.002).

**Table 1.** Demographic And Clinical Features Of The Study Population

*Features*	*Cases (n = 100)*	*Controls (n = 50)*	*t*	*p*
Age (years)	33.20 ± 7.67	31.72 ± 9.71	1.016	0.311
BMI	21.41 ± 4.35	22.56 ± 2.76	2.890	0.091
BSA (m^2^)	1.66 ± 0.19	1.68 ± 0.17	0.508	0.612
Pulse rate (beats/min)	87.04 ± 13.04	78.56 ± 6.22	4.348	0.000*
DBP (mmHg)	70.59 ± 7.39	74.60 ± 7.27	3.146	0.002*
SBP (mmHg)	111.56 ± 11.53	113.00 ± 12.98	0.687	0.493

Values are mean ± SD. BMI: body mass index; BSA: body surface area; DBP: diastolic blood pressure; SBP: systolic blood pressure. SD: standard deviation; *p < 0.05 is statistically significant.

The CD_4_ count ranged from 7.00 to 1 481.0/μl with a mean of 232.0 ± 214.8/μl.

Of the 100 cases studied, 99 had cardiac chamber dimension measurements taken. One did not because a large pericardial effusion precluded accurate measurements. Comparison of the echocardiographic dimensions between the cases and the controls is summarised in Table 2.

**Table 2.** Echocardiographic Dimensions In Cases And Controls

*Parameters*	*Cases (n = 99)*	*Controls (n = 50)*	*t*	*p*
LA (cm)	2.94 ± 0.51	2.96 ± 0.39	0.201	0.841
AO (cm)	2.71 ± 0.45	2.60 ± 0.38	0.999	0.319
RVOT (cm)	2.85 ± 0.41	2.95 ± 0.49	1.316	0.190
ERV (cm)	2.11 ± 0.43	1.91 ± 0.30	2.937	0.004*
IVS (cm)	0.97 ± 0.19	0.93 ± 0.17	1.414	0.160
LVPW (cm)	0.83 ± 0.15	0.84 ± 0.17	0.155	0.877
LVEDd (cm)	4.58 ± 0.58	4.50 ± 0.53	0.816	0.416
LVEDs (cm)	3.23 ± 0.54	2.99 ± 0.46	2.712	0.008*
LVEDd/BSA (cm/m^2^)	2.77 ± 0.35	2.69 ± 0.29	1.412	0.160
LVMI (g/m^2^)	84.33 ± 24.68	78.72 ± 23.81	42.87	0.000*
RWT	0.37 ± 0.08	0.38 ± 0.08	0.151	0.881

Values are mean ± SD. LA: left atrial diameter; AO: aortic root diameter; AOEX: aortic excursion; RVOT: right ventricular outflow tract; ERV: estimated right ventricular diameter; IVS: interventricular septum; LVPW: posterior wall thickness; LVEDd: left ventricular end-diastolic diameter; LVEDs: left ventricular end-systolic diameter; BSA: body surface area. *p < 0.05 is statistically significant.

HIV-positive patients had significantly increased LVEDs, ERV and LVMI compared with the controls. Although the LVEDd was higher in the cases, it did not reach the level of statistical significance. However left ventricular dilatation, defined as LVEDd > 5.2 cm, was significantly more in the cases than the controls (*p* = 0.03).

Comparison of left ventricular systolic and diastolic functions is summarised in Table 3. The cases had significantly reduced systolic parameters (LVFS, LVEF) and significantly increased IVRT, an important diastolic parameter, compared with the controls.

**Table 3.** Systolic And Diastolic Parameters In Cases And Controls

*Parameters*	*Cases (n = 99)*	*Controls (n = 50)*	*t*	*p*
SV	63.91 ± 24.90	66.19 ± 22.92	0.54	0.589
LVEF	64.45 ± 8.63	70.17 ± 7.08	4.05	0.000*
LVFS	29.60 ± 5.60	34.51 ± 14.61	2.95	0.004*
DT (ms)	187.82 ± 30.45	181.04 ± 16.23	1.48	0.142
IVRT(s)	88.24 ± 19.62	80.81 ± 10.81	2.48	0.015*
E	75.05 ± 16.77	77.71 ± 16.59	0.92	0.36
A	50.20 ± 11.25	51.51 ± 11.29	0.67	0.50
E/A	1.56 ± 0.49	1.55 ± 0.33	0.02	0.89
EPSS	0.45 ± 0.31	0.34 ± 0.22	2.11	0.04*

Values are mean ± SD. SV: stroke volume; LVEF: Left ventricular ejection fraction; LVFS: left ventricular fractional shortening; DT: deceleration time; IVRT: isovolumic relaxation time. E: early diastolic filling; A: atrial contraction; E/A: ratio of early (E) to late (A) diastolic filling velocities in the mitral inflow; EPSS: endpoint septal separation. *p < 0.05 is statistically significant.

Echocardiographic abnormalities were found in 78% of the cases overall compared with 16% in the controls (χ^2^ = 52.38; *p* = 0.000). The echocardiographic abnormalities are summarised in Table 4.

**Table 4.** Echocardiographic Abnormalities In Cases And Controls

*Echocardiographic abnormalities*	*Cases (n = 99)*	*Controls (n = 50)*	*χ^2^*	*p*
Pericardial effusion*	47 (47.00)	0 (0)	32.10	0.000
Systolic dysfunction	30 (30.30)	4 (8)	8.16	0.004
Diastolic dysfunction	32 (32.32)	4 (8)	9.44	0.002
Dilated left ventricle	15 (15.15)	1 (2)	4.70	0.031
Dilated cardiomyopathy	5 (5.05)	0 (0)	1.29	0.169
Isolated right-sided dilatation	1 (1.01)	0 (0)	0.12	1.000
Aortic root dilatation	1 (1.01)	0 (0)	0.12	1.000
Mitral valve prolapse	1 (1.01)	0 (0)	0.12	1.000

Values are number (%). *n = 100 cases for pericardial effusion.

Of the 100 cases studied, 30 (30%) had systolic dysfunction compared with four of the 50 controls (8%), *p* = 0.004. Twenty-five (83%) of these had mild dysfunction, while five (17%) had moderate to severe dysfunction. Of the 25 patients who had mild systolic dysfunction, 17 (68%) had a CD_4_ count less than 200/μl, while eight (32%) had a CD_4_ > 200/μl. All five (17%) with moderate to severe systolic dysfunction had a CD_4_ count < 200/μl. There was no regional wall-motion abnormality in the cases.

Furthermore, 32 (32%) of the cases had diastolic dysfunction compared with four of the controls (8%), *p* = 0.002. Of these, six (19%) had impaired relaxation; 10 (31%) had pseudonormalisation pattern, while the remaining 16 (50%) had restrictive diastolic dysfunction. Of the cases that had either pseudonormalisation or restrictive diastolic dysfunction, 15 (58%) had CD_4_ counts < 200/μl, while the remaining 11 (42%) had CD_4_ > 200/μl.

Five (5%) of the cases and none of the controls had dilated cardiomyopathy (*p* = 0.169), while one of the cases had isolated right-sided dilatation. One of the cases also had aortic root dilatation with severe regurgitation, while another had mitral valve prolapse.

**Fig. 1.**
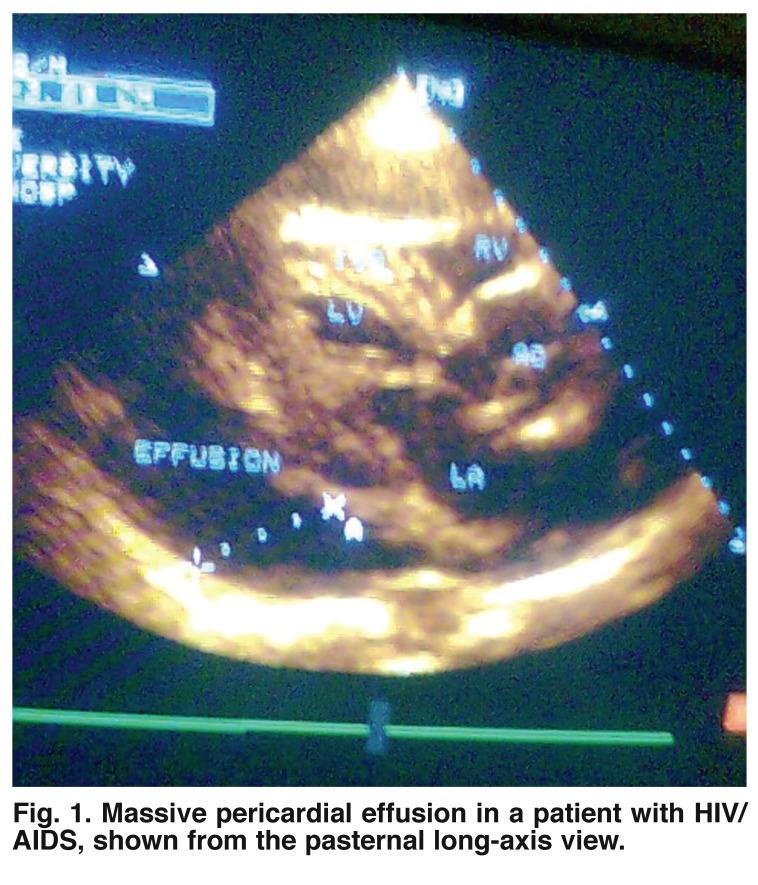
Massive pericardial effusion in a patient with HIV/AIDS, shown from the pasternal long-axis view.

Pericardial involvement was common in the cases. Of the 100 cases, 47 (47%) had pericardial effusion, while none had this in the control group (Table 4). This difference was strikingly significant (*p* = 0.000). In patients with pericardial effusion, 39 had mild effusion while eight had moderate to severe effusion, with a mean CD_4_ cell count of 125/μl (Fig. 1).

All five patients with dilated cardiomyopathy (DCM) had a CD_4_ count < 200/μl. The mean CD4 of those with DCM was 80/μl. The only patient with isolated right-sided dilatation had a CD_4_ count of 67/μl.

## Discussion

This study clearly reveals that the majority of patients with HIV infection had echocardiographic abnormalities which were clinically quiescent. This suggests echocardiography as a relevant tool for diagnosis of sub-clinical cardiac abnormalities, with the aim of instituting management early where necessary. Similar findings have been reported by other workers.^9^,^24^,^25^

Our study shows that pericardial effusion was frequently seen in our HIV-infected patients, with a spectrum ranging from asymptomatic mild effusion to severe pericardial effusion. Pericardial disease is the most frequent cardiovascular manifestation of HIV infection^24^,^25^ and it is often associated with shortened survival, independent of CD_4_ count and serum albumin values.^23^,^26^,^27^ The prevalence of pericardial disease on echocardiography in Prendergast’s study ranged from 10 to 59%,^2^ although the majority of these patients were asymptomatic. This was confirmed by our findings, where pericardial effusion was found in almost half of the patients, while only one patient had overt symptoms. With the increasing incidence of HIV infection, pericardial effusion and its attendant complications may become a major cardiac abnormality to contend with in future.

No definitive cause was determined for any pericardial effusion in this study. Determination of the aetiology of pericardial effusions in HIV-infected patients is often difficult.^22^,^23^,^26^ Pericardiocentesis is not feasible in the majority of these patients because most pericardial effusions are small,^22^,^23^,^28^ and even when indicated for the relief of tamponade, its diagnostic accuracy is said to be low.^29^

Various causative factors involved in the development of pericardial disease have been described. Tuberculosis is the commonest cause of pericardial disease in Africa,^26^,^27^ accounting for 86 to 100% of cases.^29^ Other reported causative factors include the human immunodeficiency virus itself,^2^,^30^ opportunistic infections such as cytomegalovirus,^31^ mycobacterium,^32^ cryptococcus,^33^ bacterial infections,^34^ malignancies such as Kaposi’s sarcoma,^35^ and non-Hodgkin lymphoma.^22^,^36^ It can also be part of a generalised effusive serous process involving pleural and peritoneal surfaces, which is probably a consequence of enhanced cytokine expression.^4^,^22^

The findings in this study also confirm that HIV infection was associated with left ventricular dysfunction and increased ventricular dimensions. Similar trends have been noted in other studies.^9^,^18^,^34^-^36^ The presence of ventricular dysfunction in the absence of chamber enlargement, as found in about half of those with ventricular dysfunction in our study, has also been reported in other studies.^10^,^24^,^28^ This has been posited to represent an early phase of heart muscle disease/cardiomyopathy, from which the patients eventually progress to left ventricular dilatation and dilated cardiomyopathy.^20^,^24^,^28^

Systolic dysfunction, which is a frequently documented finding in echocardiography of HIV-infected patients,^9^,^10^,^28^,^37^,^38^ was noted in about a third of our cases, signifying reduced myocardial contractility. The dysfunction was more frequent with disease progression, paralleling the reports by other workers.^9^,^10^,^28^,^37^ Systolic dysfunction is said to be an important cause of morbidity and mortality in AIDS patients.^38^ It is also posited that symptomatic heart failure will occur in approximately 6% of these patients, especially at the end stage of the disease.^26^,^35^,^39^ With this in mind, early recognition of dysfunction and institution of management may impact on the overall outcome of these patients.^9^

Diastolic dysfunction was also noted in our patients, signifying ventricular filling abnormalities due to a non-compliant ventricle.^21^ Diastolic dysfunction was also observed to be more frequent and worsening with disease progression. The findings in our study compare with the 30% prevalence noted by Danbauchi *et al*.^25^ in their work. Diastolic dysfunction has also been reported in other studies.^16^,^37^

**Fig. 2.**
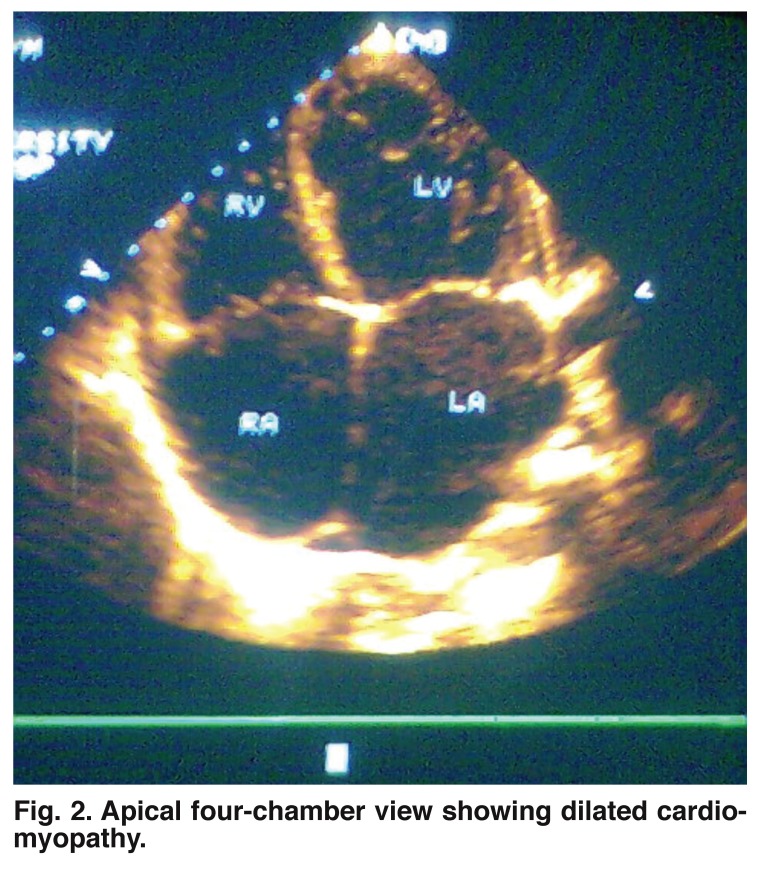
Apical four-chamber view showing dilated cardiomyopathy.

DCM is a well-documented cardiac abnormality in HIV/AIDS,^9^,^34^,^40^,^41^ and was found in 5% of our cases, with none in the control group (Fig. 2). All patients with DCM had more advanced immunosuppression with a mean CD_4_ count of 80/μl. This result correlates well with several reports that dilated cardiomyopathy in HIV is associated with advanced immunosuppression and lower CD_4_ lymphocyte counts < 100/μl.^3^,^9^,^18^,^28^,^29^ Nzuobotane *et al.*^9^ demonstrated a similar relationship between the degree of immunosuppression and the likelihood of cardiomyopathy. Interestingly, a CD_4_ count of 100/μl proved to be the important threshold in that study as well. Currie *et al*.^18^ in a similar study, reported DCM in 4% of cases, a result which parallels that of our study. In their study DCM was also strongly associated with advanced immunosuppression.

No definitive aetiologies were determined for heart muscle disease in our study. The aetiopathogenesis of cardiomyopathy remains unclear, often multifactorial.^3^,^29^,^34^,^42^ Myocarditis and direct HIV invasion of myocardial tissue are the most studied causes of dilated cardiomyopathy in HIV infection.^2^,^5^,^6^,^42^ Co-infection with other cardiotropic viruses such as Coxsackie virus, cytomegalovirus and Epstein-Barr virus have also been reported.^3^,^5^,^6^,^34^,^43^

Other causes include the cardiotoxic effect of antiretroviral drugs such as zidovudine,^26^,^34^,^44^ autoimmunity,^3^,^25^,^45^,^46^ and nutritional factors such as deficiency of selenium and other trace elements.^3^,^5^,^7^,^17^,^47^ Selenium deficiency as a cause of HIV-related heart muscle disease may be of considerable interest in Africa9 and in our study, considering that most of these patients present with multiple nutritional deficiencies, prolonged diarrhoea and wasting, which may involve selenium deficiency.^9^ Selenium supplementation has been shown to improve cardiac dysfunction in these patients.^2^,^4^,^5^,^07^,^17^

**Fig. 3.**
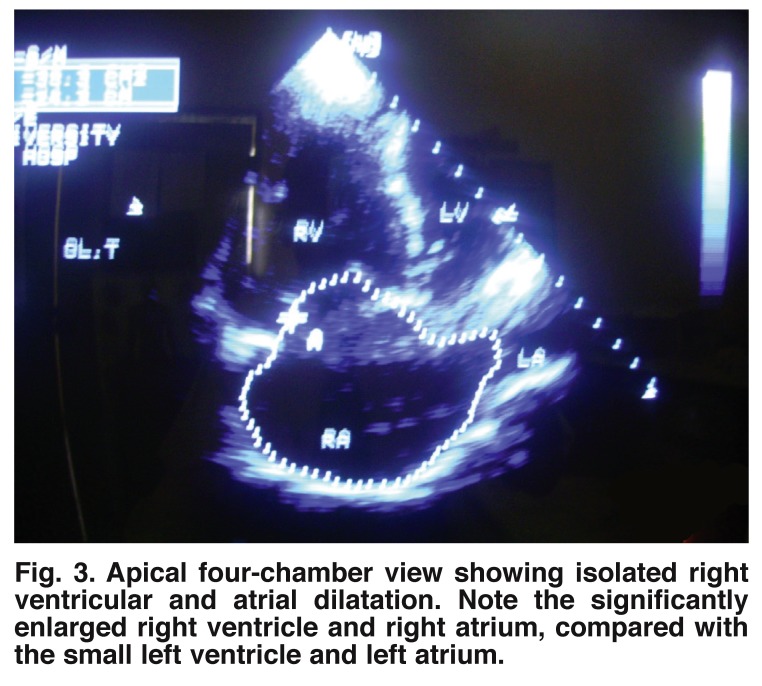
Apical four-chamber view showing isolated right ventricular and atrial dilatation. Note the significantly enlarged right ventricle and right atrium, compared with the small left ventricle and left atrium.

Isolated right heart dilatation with dysfunction was found in one of the patients in our study, who had significant pulmonary disease of over six months’ duration (Fig. 3). The very low CD_4_ count of 64/μl in this patient suggested some relationship with severe disease progression, as reported in other studies as well.^18^,^20^

**Fig. 4.**
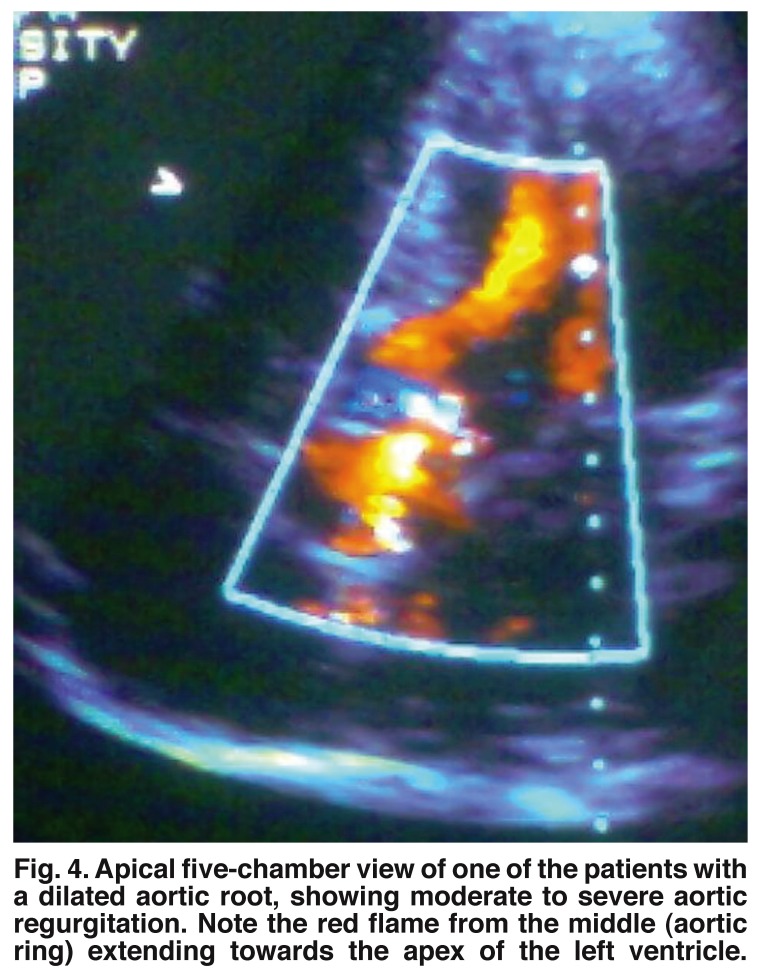
Apical five-chamber view of one of the patients with a dilated aortic root, showing moderate to severe aortic regurgitation. Note the red flame from the middle (aortic ring) extending towards the apex of the left ventricle.

One of the patients in our study, with a CD_4_ of 171/μl, had aortic root dilatation, which was associated with severe aortic regurgitation (Fig. 4). Although not common, aortic root dilatation and even aneurysm has been reported in other studies.^20^,^48^ This may be the beginning of large-vessel vasculitis of possible infective or immune-complex origin, involving the aorta and its major branches, which has been reported by other workers.^49^,^50^

Our study did not evaluate other possible co-morbidities, such as HIV-associated nephropathy and anaemia, which may be present in the patients aside from cardiovascular abnormalities. We also could not use newer methods, such as tissue Doppler, to assess diastolic function. This was unfortunate because tissue Doppler is more reliable than the method used in our study, it helps to clarify the issue of pseudonormalisation, and it is less load-dependent. Furthermore, we could not assess the prognostic implications of cardiovascular involvement in our subjects.

## Conclusion

In view of the high frequency of cardiac abnormalities detected by echocardiography in the HIV/AIDS patients in our study, it is suggested that HIV-positive patients should have a careful initial and periodic cardiac evaluation to detect early involvement of the heart in the HIV disease.
